# Nanovesicles as Potential Carriers for Delivery of Antiviral Drugs: A Comprehensive Review

**DOI:** 10.2174/0115672018313783240603114509

**Published:** 2024-06-06

**Authors:** Sabitri Bindhani, Amit Kumar Nayak

**Affiliations:** 1 Department of Pharmaceutics, School of Pharmaceutical Sciences, Siksha ‘O' Anusandhan (Deemed to be University), Bhubaneswar, Odisha, 751003, India

**Keywords:** Nanocarriers, nanovescicles, nanovesicle manufacturing, liposomes, transferosomes, antiviral drugs, drug delivery, viral diseases

## Abstract

Different nanocarriers-based strategies are now extensively being used as an important strategy for improving drug efficacy and responsiveness, reducing toxicity issues related to drugs and harmful side effects, and overcoming the numerous significant difficulties related to absorption and bioavailability. Amongst different nanocarriers, nanovesicles are excellent and versatile systems for effectively delivering therapeutic agents, targeting ligand distribution and location. Nanovesicles are nanosized self-assembling spherical capsules with an aqueous core and one/more lipid(s) layers. Several synthetic nanovesicles have been developed and investigated for their prospective uses in delivering drugs, proteins, peptides, nutrients, *etc*. Important procedures for nanovesicle manufacturing are thin-film hydration, unshaken method, ethanol injection, ether injection, proliposomes, freeze-drying, hot method, cold method, reverse-phase evaporation, and ultrasonication. Liposomes, liposomes, ethosomes, exosomes, and transferosomes (elastic vesicles) are the nonvesicular candidates extensively investigated to deliver antiviral drugs. This review article comprehensively overview different nanovesicles, their compositions, manufacturing, and applications as potential carriers for effectively delivering different antiviral drugs to treat viral diseases.

## 1 INTRODUCTION

The primary source of high mortality and continuously magnified adverse socioeconomic effects, viral infections are the primary reason for health problems globally [1, 2]. The impacts of viruses on human health can range from minor to potentially fatal. Many aspects (including the prevalence of co-morbidities, the wide range of viral diseases, social and psychological issues with financial implications, and the variety of treatments and immunizations that can be used in the evaluation) make it difficult to estimate the overall impact of viral infections. Major pathogenic viruses, like hepatitis viruses, human immunodeficiency virus (HIV), herpes simplex virus (HSV), corona viruses, human papilloma virus (HPV), *etc*., cause a significant amount of human death and morbidity [3, 4]. A deadly outbreak caused by the new corona virus (nCoV) has declared about 2.1 lakh lives globally, with benefits given. In India, 19 Nipah virus infections were reported in 2018, with 17 of them resulting in fatalities. Since 2001, the Nipah virus has caused a mortality rate in India that ranges from 68 to 100% [5]. HIV and Hepatitis C (HCV) are both chronic viral infections for which there are no reliable vaccinations. Between 2014 and 2016, a pandemic of major Ebola virus disease killed 11,315 people out of 28,616 cases in West Africa [6]. Many factors have been identified as important risks for viral infections, including the availability of water, sanitation services, and weather conditions, lifestyle factors such as alcohol consumption, geographic location, and diagnosis and treatment options such as blood donation, surgery, and vector transmission [7].

One efficient strategy for enhancing the immune system's defenses against pathogens is vaccination, which often lowers the burden of infectious diseases and lowers mortality and morbidity rates. To control a global pandemic and stop the proliferation of an epidemic, different new methodologies in vaccine research are essential in health emergencies [8, 9]. Antiviral drugs are efficient against virus infections, and their effectiveness is typically constrained by issues like poor solubility, less permeability, untargeted release, reduced oral bioavailability, unfavourable side effects, antiviral resistance, *etc* [10]. High antiretroviral activity therapy (HAART), which includes three antiretroviral (ARV) drugs, is used to treat HIV/AIDS [11]. Even though ARV medications are used to treat some infections, complete recovery has not yet been achieved because of several drawbacks, such as impaired absorption, restricted regulated administration, moderate water solubility, *etc*. Viruses significantly impair the host's immune system, and most medicines are clinical, so eradicating viral infection may be unattainable due to the complexity of viruses. It is challenging to develop a particular medicine that can identify the virus without hurting healthy cells because the mechanism of viral infections depends on the properties of causative viruses and the host cells. Another key component is the formulation development for antiviral drugs, which comprises changes in the physico-chemical as well as biopharmaceutical characteristics of antiviral drugs. The pharmacokinetics and bioavailability properties are improved by repurposing the drug or employing nanotechnology, and the delivery systems for administering antiviral drugs are improved [12, 13]. These drug delivery methods for antiviral drugs are made from synthetic or organic components. Nanomaterials are excellent therapeutic agents for viral infections because of their distinctive physicochemical characteristics. Several types of nanomaterials, including nanoparticles, dendrimers, nanoemulsions, nanosuspensions, nanoliposomes, nanogels, and nanospheres, have been discovered for the drug administration of antiviral drugs [13, 14]. These nanocarriers-based strategies are crucial for improving drug efficacy, and responsiveness, reducing toxicity and harmful effects, and surmounting the numerous significant difficulties related to absorption and bioavailability [15]. This review article presents a comprehensive review of different nanovesicles, their compositions, manufacturing and applications as potential carriers for effective delivery of different antiviral drugs for the treatment of viral diseases.

## 2 METHODOLOGY OF REVIEW

The searching of reported literature for the current review was carried out using different popular scientific databases like Science Direct, Scopus, Web of Science, Google Scholar, and PubMed. The keywords employed for searching the literature sources comprise nanomedicines, nanovesicles, liposomes, transferosomes, niosomes, ethosomes, antiviral drugs, and antiviral therapy. This review covers all published research on the current topic to date.

## 3 NANOVESICLES

The most significant issue in biotechnology and nanomedicine is determining how to advance biomaterials and nanomaterials, which actively transport entrapped drugs to particular cells or tissues when the effect of increased permeability and retention (EPR) is inadequate [16]. In recent decades, a few examples of biocompatible organic and inorganic nanosystems, including liposomes, nanoparticles (MSNPs), hydrogels, mesoporous silica, and gold nanoparticles (AuNPs), niosomes, ethosomes, transferosomes, and pharmacosomes that have been developed and researched as delivery systems to target tumor cells [17, 18]. In 1965, the late Alec Bangham and colleagues presented swelling phospholipid systems, which inspired model membrane systems [19]. Numerous enclosed phospholipid bilayer structures made of single bilayers first referred to as “Banghosomes” and later called “liposomes,” were described briefly. Gregory Gregoriadis discovered that liposomes for entrapping drug molecules and can be employed as drug-delivery carriers [4]. Amongst different nanocarriers, nanovesicles are excellent versatile carrier systems, widely used for effective delivery of drugs, biomolecules, and targeted ligand distribution and location [20-23]. Nanovesicles have been found as feasible nanocarriers in drug delivery due to their inherent biological features obtained from polymeric molecular self-assemblies [21, 24].

### 3.1 Classification

Nanovesicles are generally nanosized self-assembling spherical capsules having an aqueous core and one or more layers of lipid(s) [8]. Naturally, nanovesicles are tiny sacs that transport chemical molecules secreted by cells as a means of communication. A number of synthetic nanovesicles namely; liposomes, transferosomes, exosomes, niosomes, ethosomes, cubosomes, aquasomes, emulsomes, bilosomes, ufasomes, phytosomes, erythrosomes, pharmacosomes, virosomes, enzymosomes, colloidosomes, sphingosomes, discomes, *etc*., have been developed and researched for their prospective uses for delivery of drugs, protein and peptides, nutrients, *etc* [21].

Amongst several nanovesicles, liposomes are lipidic vesicular structures composed of an aqueous core encased by one/more lipid bilayer membranes of synthetic, semisynthetic, or natural phospholipids [25]. The distinctive structural feature of liposomes allows them to load both hydrophobic and hydrophilic drug candidates [26]. Niosomes (non-ionic surfactant vesicles) comprise microscopic lamellar bilayer structures produced upon combining non-ionic surfactant(s) with cholesterol [27]. Niosomes form closed bilayer vesicles in the aqueous media on the basis of amphiphilic characteristics employing some energy, such as heat, physical agitation, *etc*., to produce niosomal structure. Transferosomes (deformed liposomes) are elastic-natured vesicular systems, where at least one aqueous compartment is encased by a lipidic bilayer and an edge activator [28]. Ethosomes are another category of vesicles containing phospholipids, ethanol and water [29, 30]. Phytosomes are lipid-compatible plant-based extract molecular complexes [29]. Pharmacosomes are colloidal dispersions containing pharmaceuticals, which are usually covalently linked to lipids [31]. Virosomes are lipid bilayers made of lipids derived from retroviruses that have been injected with virus glycoproteins [32, 33]. Enzymosomes are liposomal units, which are formed when an enzyme is co-valently attached to the liposomal surface [34]. Ufasomes (unsaturated fatty acid liposomes) are vesicles, where long-chain fatty acids and soap are combined to form vesicles [35]. The oral delivery of vaccinations is made possible *via* bilosomes, which are bile salt-based delivery systems [31]. Aquasomes are spherical nanosized vesicles that are nanosized and used to deliver drugs and antigens [36]. Emulsomes are lipid-based nanosized vesicles with a polar group and a lipid assembly [37]. Cubosomes are lipid-based nanovesicles of bicontinuous cubic phases divided by a lipid layer into two distinct, continuous, but non-intersecting hydrophilic zones [38]. Sphingosomes are vesicles comprising an aqueous core surrounded by means of a lipidic bilayer membrane composed of sphingolipid(s) [39]. Colloidosomes are nanosized hollow shells of colloidal dispersions whose permeability and flexibility are adjusted during manufacturing [40]. Human erythrocyte cytoskeletons undergo chemical cross-linking coat lipid-based structures called erythrosomes [41]. Discomes are substantial disk-shaped structures created when a specified amount of surfactant is added to a mixture of vesicular dispersions [42].

### 3.2 Compositions

Different synthetic varieties of nanovesicles possess distinctive compositional features.

#### 3.2.1 Liposomes

Liposomes are colloidal, microscopic, concentric bilayered vesicles containing an aqueous core surrounded with phospholipid bilayer membranes [25]. These phospholipids include phosphatidylcholine, phosphatidylglycerol, phosphatidylserine, phosphatidylinositol, *etc*. Phosphatidylethanolamine and phosphatidylcholine are the neutral phospholipids, whereas phosphatidylserine and phosphatidylglycerol are the anionic phospholipids. The composition of liposomes contains cholesterol, which amplifies the bilayer’s stability and membrane fluidity. As the phospholipid bilayers are the most common components of liposomes, they are structurally comparable to the cell membranes in their fundamental composition and function. These characteristics have greatly enhanced the demand for liposomes as drug-delivery vehicles and as cell-mimicking models in research involving membrane functions, drug-cell interactions, and internalization procedures. In addition to this, it decreases the aqueous-soluble molecules’ permeability across the membrane [43]. Because of their unique structural features, they entrap both hydrophobic and hydrophilic drug candidates. The hydrophobic drug candidates are loaded in the lipid bilayer; hydrophilic drug candidates are loaded into their hydrophilic core [26].

#### 3.2.2 Niosomes

Niosomes have structural similarities with liposomes. The vesicles contain a bilayer of non-ionic surfactants (*e.g*., Span 40 and 60) and cholesterol [27]. The non-ionic surfactants start to align themselves so that the aqueous-soluble ends are directed toward one another and the hydrophilic ends of the non-ionic surfactant point outward in all directions to stabilize the bilayer. Its lipid membrane can either be unilamellar or multilamellar, which are more stable than liposomes. In most cases, hydrophilic-lipophilic balance (HLB) value is used to guide the surfactant selection. Non-ionic surfactant molecules tend naturally to arrange themselves so that hydrophilic ends point outwards, whereas hydrophobic ends face each other to produce the bilayer structure. Because of the incorporation of nonionic surfactants as composition materials, the size and charge of niosomes are both enhanced, which further improves their encapsulation efficiency.

#### 3.2.3 Transferosomes

Transferosomes are elastic vesicles containing phospholipids and edge activators. They are more favourable than conventional liposomes and niosomes due to their excellent elasticity and ability to penetrate wide pore sizes [28, 44]. Transferosomes are substantially more stable than conventional liposomes, and the self-developed bilayer lipid vesicle can survive external strain [45]. In the preparation of transferosomes, edge activators are used to enhance the elasticity of the membrane and help in the deformation of vesicles. Phospholipids are the main lipidic bilayer compositions of transferosomes presenting the vesicle stability. Therefore, the incorporation of membrane-stabilizing agents like phospholipids and destabilizing agents is essential for non-rigid or elastic vesicles (transferosomes). Transferosomes are produced using a variety of edge activators, including sodium cholate, sodium oleate, sodium deoxycholate, diacetyl phosphate, dipotassium glycyrrhizinate, Tweens, Spans, *etc* [46]. Two examples of lipid bilayer raw materials that prepare transferosomes are soy lecithin and soy hydrolyzed phosphatidylcholine [47].

#### 3.2.4 Ethosomes

Ethosomes are soft-natured and novel vesicular carrier systems that are specifically designed for improved drug delivery (*e.g.,* transdermal distribution). They are more frequently utilized in cosmetic preparations because of the properties of their composition. Ethosomes are soft and flexible vesicular carriers containing high concentrations of ethanol (20–45%), designed for improved delivery of drugs [29]. By means of interacting with the polar heads of lipids, they are frequently employed to lower the melting points of lipids in the stratum corneum, to enhance the lipid fluidity and to improve the cell membrane permeability. Ethosomes are predominantly composed of phospholipids, for example, soybean phosphatidylcholine. Moreover, propylene glycol is usually employed, which also improves penetration [48]. By incorporating penetration enhancers like labrasol, transcutol, and PEG 400 for the ethanol in their phospholipid bilayer, they overcome the drawbacks of ethosomes.

### 3.3 Manufacturing

Several manufacturing procedures are used to produce nanovesicles based on vesicle types, drugs, scalability, and lamellarity.

#### 3.3.1 Thin Film Hydration

The common approach for preparing nanovesicles is the thin film hydration process. It requires simply withdrawing the solvent from a flask with a round bottom, adding the dispersion medium, and stirring to generate a thin hydrophobic layer. However, the formation of nanovesicles begins to be heterogeneous with continued agitation. It must be extruded through a polycarbonate membrane to make it homogeneous [49].

#### 3.3.2 Nonshaken Method

This approach involves exposing the film to a stream of nitrogen-rich water to hydrate it, after which it can begin swelling in a liquid suspension devoid of shaking operation. This approach yields large, in unilamellar vesicles [50].

#### 3.3.3 Proliposomes

Hydration occurs immediately following the absorption of vesicles. Here, phospholipid materials are treated on finely divided particles with an air support, like sorbitol or sodium chloride. These lipid-coated powders (proliposomes) are mixed with water only when necessary to prepare a suspension of multilamellar vesicles [51].

#### 3.3.4 Freeze-drying Method

It is involved in preparing a homogeneous lipidic dispersion in aqueous soluble carrier molecules. To prepare the transparent and isotropic monophase solution, aqueous soluble carriers, like sucrose and liposome-forming lipids, are solubilized in alcohol/water cosolvent systems. Afterward, the above solution is sterilized and transferred into vials for freeze-drying. In an investigation, the drying process is carried out in a laboratory freeze-drier [51]. The ratio of lipid to carrier is the main reason influencing the sizes and polydispersity of the liposome formations based on an analysis of the method's many characteristics.

#### 3.3.5 Ethanol Injection Method

This method is primarily employed to make tiny needles. To prevent drying of the alcohol, the drugs and soy phosphatidylcholine are incorporated into ethanol in a closed glass container. The mixture is then stirred continuously, and distilled water is syringed into the mixture in a streamlined motion [52].

#### 3.3.6 Ether Injection Method

Niosomes are usually prepared using this method of vesicle preparation, which is different from the ethanol injection. The solvent is expelled from the liposomal product by heating ether, which is insoluble in aqueous phase. This method entails infusing ether-lipid solutions into a warmed aqueous environment above the boiling point of ether. The ether evaporates upon contact with the water. Compared to ethanol injection, this method of vesicle preparation has the benefit that the product is solvent-free, facilitating the manufacturing procedure to be performed for more extended periods and producing concentrated liposomal constituents with increased entrapment [53].

#### 3.3.7 Hot Method

This technique is usually used to prepare ethosomes. In the hot method process, the drug is dissolved in a solution of ethanol-propylene glycol before being added to phospholipid dispersion in water at 40°C. Following a thorough mixing, it is then sonicated at a temperature of 4°C for 3 cycles of 5 min each; with a 5 min break in-between each cycle. Then, the mixture is well homogenized by means of a high-pressure homogenizer to produce nanosized ethosomes [52].

#### 3.3.8 Cold Method

This method is well-known as one of the most popular and commonly applied manufacturing procedures for ethosomes. Drug, phospholipids, and other lipidic components are dissolved in ethanol with constant stirring at room temperature. The obtained drug-lipids mixture is heated up to 30°C, using temperature controlled water bath, and after being heated, water is adjoined to the mixture and then well-stirred for a period of 5 min in a closed vessel. If necessary, the ethosomal formulation sizing may need to be reduced through an additional sonication process or extrusion process. The formulation must safely be refrigerated [52].

#### 3.3.9 Heating Method

The heating method is effective for the production of nanovesicles, specifically powder-inhalable nanovesicles. In this method, nanovesicles are produced in an environmentally friendly manner without the use of solvents or detergents [54]. In brief, the drug, cholesterol, and surfactants, are added to the aqueous. The solution is prepared by stirring and heating the aqueous phase. Afterwards, 3% *v*/*v* polyol (for example, glycerol) is added to the solution. The heating method does not use any toxic organic solvent [55].

#### 3.3.10 Reverse-phase Evaporation

This manufacturing procedure involves the evaporation of organic solvents to form a lipidic layer at reduced pressure. After nitrogen is removed from the system, the lipidic materials are redissolved in a subsequent organic phase containing ether(s) (isopropyl ether and/or diethyl ether). Adding an aqueous buffer leads to the development of unilamellar as well as oligolamellar vesicles. The organic solvents evaporate, keeping the system under constant nitrogen pressure. These vesicles contain 30 times more aqueous volume to lipid ratios than multilamellar and sonicated preparations. Most notably, the vesicles contain a significant amount of the aqueous phase, efficiently encapsulating even large macromolecular assemblies [51].

#### 3.3.11 Ultrasonication

An alkaline step treats the surfactant-cholesterol mixtures in glass beakers. After that, a probe is continually used to stir the solution for a predetermined period. The resulting vesicles have a thin, uniform, and unilamellar form. The comparable vesicle sizes of niosomes are typically larger than that of conventional liposomes. This technique is useful to prepare niosomes larger than 100 nm in diameter [51].

## 4 OVERCOMING THE CHALLENGES OF CONVENTIONAL SYSTEMS FOR THE DELIVERY OF ANTIVIRAL AGENTS

The administration of antiviral agents *via* different conventional dosage forms is associated with several issues, including limited bioavailability, toxicity issues, drug resistance, generalized targeting, and short half-lives of antiviral agents. Nanomedicine, or the use of nanotechnology in diagnostic and therapeutic settings, is the process of downsizing materials to the nano-range [56]. A novel category of pharmaceutical nanomaterials known as nanopharmaceuticals exhibits the benefits of a high surface/volume ratio, compact sizes, and favourable surface chemistry issues. Also, antiviral nanocarriers can be administered intravenously without the risk of being retained by pulmonary capillaries [57]. Different challenges of conventional delivery of antiviral drugs are presented in Fig. (**1**).

## 5 DELIVERY OF ANTIVIRAL DRUGS USING NANOVESICLES

Advancements in drug delivery technology are undergoing a revolution in the era of nanobiotechnology. Compared to conventional therapy, nanobiotechnology significantly improved medication pharmacodynamics and pharmacokinetics by improving drug-specific targeting and extending drug bioavailability in the body [58]. A particular emphasis is being placed on delivering antiviral medicines using various nanocarriers. In addition, employing the right nanocarriers made it possible to deliver poorly soluble antiviral medications, increase drug stability, promote pharmacokinetics, and decrease unfavourable immune activation [59, 60]. Different nanovesicles for antiviral drug delivery and their potential advantages are presented in Fig. (**2**).

### 5.1 Liposomes

These are synthetic vesicles encapsulated in a phospholipid bilayer of lipids [61]. Within liposomes, many insoluble and aqueous-soluble drugs can be entrapped and solubilized into the bilayer as well as an aqueous core because of the presence of both hydrophilic and hydrophobic moieties [62]. Their size impacts the drug encapsulation and half-lives of drugs [63, 64]. PEGylation of liposomes helps to maintain their half-lives and the transport of drugs to the action site [65]. The drug's permeability, stability, bioavailability, and bioaccumulation at HIV reservoir locations are all improved by the liposomal delivery strategy. Moreover, liposomes are easily surface-modifiable, which is effective for targeted drug delivery.

In a work, Shen and Tu (2007) designed ophthalmic ganciclovir-loaded liposomes and evaluated their ocular pharmacokinetics [65]. These ophthalmic liposomes were formulated by reverse-phase evaporation. The transcorneal ganciclovir permeability (*in vitro*) of ganciclovir-loaded liposomes was 3.90-fold higher than that of ganciclovir solution. Any variation was not noticed in the precorneal ganciclovir elimination rate from ganciclovir-loaded liposomes *vs*. ganciclovir solution, *in vivo* administration in Albino rabbits. These ganciclovir-loaded liposomes showed a 1.70-fold elevated ganciclovir concentration in aqueous humor than that of ganciclovir solution. Korvasová *et al*. (2012) designed cidofovir-loaded stable cationic liposomes using some new cationic lipids, which were synthesized on the basis of spermine linked to different anchors of hydrophobic character [66]. *In vitro* toxicity of these cidofovir-loaded cationic liposomes was carried out on the Madin-Darby bovine kidney (MDBK) cells (generally used as a cell-line model for viral activity) and some cancer cell-lines. The results of *in vitro* toxicity demonstrated no toxicity by the drug-free cationic liposomes and cidofovir-loaded cationic liposomes. The delivery of loaded cidofovir and calcein (a fluorescence marker) into cells has been attained because of the large amount of internalization of cationic liposomes as confirmed by fluorescent microscopy and analyzed by flow cytometry. The significantly improved antiviral action of cidofovir was found in the case of cidofovir-loaded cationic liposomes.

Law *et al*., (2000) formulated acyclovir-loaded ocular liposomes for enhanced corneal penetration and absorption of loaded acyclovir [67]. In this research, both *in vitro* and *in vivo* evaluations were performed to study corneal penetration and absorption of loaded acyclovir, respectively, from acyclovir-loaded liposomes. The corneal penetration (*in vitro*) result revealed that the cationic acyclovir-loaded liposomes were capable of producing a lower acyclovir penetration rate as compared to that of anionic acyclovir-loaded liposomes and acyclovir solution. On the other hand, *in vivo* corneal absorption results revealed that the acyclovir absorption rate from cationic acyclovir-loaded liposomes was found higher as compared to that of anionic acyclovir-loaded liposomes and acyclovir solution. The concentration of acyclovir in the cornea after ocular administration of cationic acyclovir-loaded liposomes demonstrated a greater rate of corneal deposition of acyclovir in comparison to that of anionic acyclovir-loaded liposomes and acyclovir solution.

Naderkhani *et al*., (2014) developed mucoadhesive liposomes to increase the permeability of loaded acyclovir [68]. These acyclovir-loaded liposomes were formulated using egg phosphatidylglycerol and egg phosphatidylcholine. The negatively charged acyclovir-loaded liposomes made of egg phosphatidylcholine/egg phosphatidylglycerol could entrap more acyclovir compared to that of the neutral charged acyclovir-loaded liposomes made of egg phosphatidylcholine. The entrapment of acyclovir within liposomes displayed a significant enhancement in *in vitro* permeability of loaded acyclovir, than that of the acyclovir aqueous solution. The neutrally charged acyclovir-loaded liposomes demonstrated higher acyclovir permeability (*in vitro*) compared to that of the charged acyclovir-loaded liposomes made of egg phosphatidylcholine/egg phosphatidylglycerol. The acyclovir-loaded liposomes were coated with Carbopol to make mucoadhesive, which increased the acyclovir entrapment within the neutral-charged acyclovir-loaded liposomes. The Carbopol-coating onto these acyclovir-loaded liposomes demonstrated a significant enhancement in *in vitro* permeability of loaded acyclovir in the case of acyclovir-loaded liposomes made of egg phosphatidylcholine/egg phosphatidylglycerol and acyclovir-loaded sonicated liposomes made of egg phosphatidylcholine. Both these acyclovir-loaded liposomes demonstrated the maximum *in vitro* permeability of loaded acyclovir compared to that of all tested formulations. Ramana *et al*., (2012) investigated the release rate of saquinavir-loaded PEGylated liposomes in mammalian cells [69]. According to the results of *in vitro* tests, saquinavir-loaded PEGylated liposomes exhibited a sustained saquinavir-releasing profile and lower cytotoxicity than non-PEGylated liposomes.

Asasutjarit *et al*. (2020) designed and evaluated transferrin-conjugated liposomes containing ganciclovir *via* the reverse-phase evaporation technique for topical instillation and intravitreal injection [70]. The physicochemical properties of transferrin-conjugated liposomes of ganciclovir were found to be affected by the composition of liposomes. The optimized formulation of transferrin-conjugated liposomes of ganciclovir exhibited a negative zeta potential and nanometer-ranged sizing (<100 nm). The *in vitro* cytotoxicity results on the human retinal pigment epithelial cells (ARPE-19 cells) demonstrated that the optimized formulation of ganciclovir-loaded transferrin-conjugated liposomes was safe. These transferrin-conjugated liposomes containing ganciclovir were taken up by ARPE-19 cells *via* transferring receptors-mediated endocytosis and exhibited an inhibitory action on cytomegalovirus in the infected cells. Hence, the optimized transferrin-conjugated liposomes containing ganciclovir could be used for targeted ganciclovir delivery to the retina to treat cytomegalovirus retinitis. In another research, a combination of two antiretroviral drugs, namely, saquinavir and nevirapine, was conjugated with stealth anti-CD4 conjugated immunoliposomes [71]. These immunoliposomes exhibited significant inhibition of viral proliferation in comparison to those of free drugs alone (saquinavir and nevirapine). The immunoliposomes conjugated with anti-CD4 might have produced a selective delivery of saquinavir and nevirapine, to the HIV-infected cells *via* the CD4 receptor. Therefore, this approach with the use of stealth anti-CD4 conjugated immunoliposomes might efficiently inhibit the viral proliferation with lower concentrations of saquinavir and nevirapine, probably decreasing the toxicity as well as resistance issues related to these two antiretroviral drugs. Some other studies on liposomal formulations for antiviral drug delivery are summarized in Table **1**.

### 5.2 Niosomes

Niosomes are being delivered *via* topical applications, including transdermal and ocular drug delivery. These are more consistent, stable, and economical as compared to liposomes, and niosomes [72-88]. Compared to liposomes, niosomes contain a higher proportion of the drug-loaded substance. In addition, niosomes offer a more excellent residence time in blood, and thus, this allows less frequent dosing. In research, zidovudine was loaded in niosomes to treat AIDS [89]. In the treated rabbit serum, the niosomal formulations showed a prolonged half-life of zidovudine. Ruckmani and Sankar (2010) formulated zidovudine-loaded niosomes for effective AIDS therapy [90]. In this research, zidovudine-loaded niosomes were optimized by varying cholesterol, Span and Tween contents as composition-related variables; whereas the influence of process variables, such as sonication time, hydration time, charge-inducing agent, rotational and centrifugation speed of evaporation flask on the zidovudine entrapment and releasing of zidovudine from zidovudine-loaded niosomes was investigated. Zidovudine-loaded niosomes formulated using Tween 80 were found to be highly zidovudine entrapped. In addition, the incorporation of dicetylphosphate improved the zidovudine release (88.72%) over a prolonged time for a longer time (> 12 h). Jacob *et al*. (2017) prepared acyclovir-loaded niosomes *via* coacervation phase separation and these acyclovir-loaded niosomes were transferred to gel formulations for skin delivery [91]. They also optimized acyclovir-loaded niosomal gel by 3^2^ factorial design, where various ratios of cholesterol, phospholipids and nonionic surfactants were varied. The drug entrapment of these niosomes was found to be influenced by the alteration of surfactant concentration. The *in vitro* releasing of acyclovir from these niosomes was found to be impacted by the use of surfactant combinations, especially by the cholesterol to lecithin ratio. In *ex vivo* permeation evaluation, the result indicated a substantial variation in permeation fluxes and also was found to be impacted by the niosomal composition. In addition, the optimized acyclovir-loaded niosomal gel indicated greater drug diffusion into the skin-layers to form a depot than that of the commercially available acyclovir cream (as control). The plasma drug level suggested a lower systemic *in vivo* exposure of acyclovir for acyclovir-loaded niosomes.

Using the reverse-phase evaporation method, Akhter *et al*. (2011) designed ganciclovir-loaded mucoadhesive niosomal dispersion for ocular administration [92]. These prepared niosomes were round-shaped with 23-200 nm of sizing, which were found to be nonirritant and nontoxic. Mehta and Jindal (2015) developed nevirapine-loaded niosomes using tyloxapol (a biological surfactant) and variable concentrations of cholesterol [93]. Formulations of nevirapine-loaded niosomes prepared using a 1:0.1 surfactant to cholesterol molar ratio exhibited the maximum stability with optimal hydrophobicity. The drug release evaluation suggested that the nevirapine efflux was found to be sustained with a depot effect and lessened the nevirapine-releasing fluctuations. Zidan *et al*. (2011) designed tenofovir-loaded niosomes with the use of a high-pressure homogenizer [94]. The tenofovir released from tenofovir-loaded niosomes was found to be significantly impacted by the factors related to the composition of niosomes rather than the physical ones. These tenofovir-loaded niosomes might be used for anti-HIV therapy in pediatric patients.

Patel *et al*. (2012) developed niosomal gel *via* a thin-film hydration technique for transdermal lopinavir delivery [95]. The formulation optimization was carried out with varied quantities (molar) of both cholesterol and Span 40 to attain desirable properties of niosomes. The optimized niosomal gel containing lopinavir exhibited high drug entrapment with minimal sizing of vesicles. The obtained results of histopathological studies suggested a better safety for the optimized lopinavir-loaded niosomes as compared to that of lopinavir-loaded ethosomes. In male Wistar rats, the bioavailability evaluation results demonstrated significantly higher *in vivo* lopinavir absorption *via* the transdermally applied niosomal gel of lopinavir in comparison to that of the oral suspension of lopinavir. Hashim *et al*., (2010) developed ribavirin-loaded niosomes for liver targeting, which were prepared *via* thin-film hydration, where cholesterol, dicetyl phosphate, and Span 60 were used in different molar ratios [96-104]. These ribavirin-loaded niosomes prepared using a 4:2:1 molar ratio of Span 60, cholesterol, and dicetyl phosphate demonstrated a significantly higher drug (ribavirin) entrapment and more sustained release of ribavirin as compared to those of other niosomal formulations prepared with other molar ratios. This above-discussed niosomal formulation was chosen for a liver targeting study, *in vivo*. The ribavirin-loaded niosomal formulation with a 30 mg/kg ribavirin as a single dose was administered by intra-peritoneal injection into rats. The results exhibited that the ribavirin-loaded niosomal formulation (prepared using 4:2:1 molar ratio of Span 60, cholesterol, and dicetyl phosphate) significantly augmented the liver concentration of ribavirin (6-fold increment) as compared to that of free ribavirin solution. Some other studies on niosomal formulations for antiviral drug delivery are summarized in Table **2**.

### 5.3 Ethosomes

These are usually utilized for transdermal drug delivery administration. They are employed to deliver antiviral drugs because they enable improved drug penetration and reduce skin irritability. Several investigations have been reported on the design and delivery of antiviral agent-loaded ethosomes [105]. For instance, zidovudine, an anti-HIV drug, was loaded in ethosomes for enhanced transdermal administration [106]. In this research, the skin penetration rate of zidovudine-loaded ethosomes was evaluated. The extent of skin permeability was evaluated using fluorescence microscopy. The findings revealed that zidovudine-loaded ethosomes exhibited the highest penetration rate as compared to others.

Patel *et al*. (2012) designed a lopinavir-loaded ethosomal carrier for transdermal delivery of lopinavir. The lopinavir-loaded ethosomal carrier exhibited good *ex vivo* skin permeation of lopinavir [95]. The bioavailability *via* the transdermal delivery was tested, *in vivo*, in male Wistar rats. In male Wistar rats, the *in vivo* bioavailability evaluation results demonstrated significantly higher lopinavir absorption *via* the transdermally applied lopinavir-loaded ethosomal carrier in comparison to that of the oral suspension of lopinavir. In another research, Dubey *et al*. (2010) investigated the indinavir delivery *via* ethosomes with transdermal administration [107]. In this work, optimal ethosomal formulations were formulated with ethanol (45%) and soya phosphatidylcholine (3%), which exhibited high drug entrapment efficiency (96.71% ± 1.4%) and 147 ± 4.5 nm vesicle size. The high drug entrapment efficiency of indinavir-loaded ethosomal formulation might be due to the enhanced interaction of ethanol with lipophilic natured indinavir. The transdermal permeation of indinavir was found significantly increased by the indinavir-loaded ethosomes than that of indinavir solution, ethanolic solution of indinavir and indinavir-loaded conventional liposomes. The percutaneous flux of indinavir in the case of the indinavir-loaded ethosomes was measured at 27.2 ± 4.2 mg cm^-2^/h.

A different investigation investigated the transdermal delivery of acyclovir *via* ethosomes [108]. In this research, acyclovir prodrug was loaded in ethosomes to compare their effectiveness. The results revealed that these ethosomes had a significantly higher acyclovir concentration in the epidermal layers than the free prodrug. Another antiviral drug, lamivudine, was encapsulated within ethosomes and liposomes for transdermal delivery [109-115]. These lamivudine-loaded ethosomes presented a 25-fold greater flux across skin than the solution of free lamivudine. The effect of ethosomes disrupting the stratum corneum and the ethosomes' flexibility might be the leading causes of increased skin permeation. Some other studies on ethosomal formulations for antiviral drug delivery are summarized in Table **3**.

### 5.4 Exosomes

Exosomes are external lipid bilayer-made nanovesicles produced by cells [116]. These are in large quantities that occur in body fluids like saliva, blood, urine, *etc*., responsible for transporting cell-specific components, like and proteins, lipids, and genetic materials. These are picked up by reprogrammed distant cells depending on the specific cargo provided [117]. In general, exosomes are released by eukaryotic cells and are known to typically for transporting cell-specific components to the distant cells as inter-cellular messengers, which are essential for viral infections. During infection due to influenza virus, exosomes change composition, causing them to express antiviral proteins, helping to trigger inflammatory responses and preventing the virus from interacting with pulmonary cells [118, 119]. Exosomes are also highly selective in their localization and possess a special ability to interact with target cells. Owing to their inherent reaction to viruses and their infections, exosomes present great promise as a kind of nanovesicular system for the delivery of antiviral agents.

Bedford *et al*. (2020) conducted an *in vivo* experiment on airway exosome releasing by employing a mouse model of influenza virus infection [120]. In this research, the research findings demonstrated that the infection affected the protein composition of exosomes. In this experiment, pulmonary inflammation was generated by intravenously administering exosomes from infected to healthy mice. In this investigation, exosomes carrying antigens were attached to the surfaces of healthy cells, preventing them from becoming infected. According to this study, these exosomes may be used to treat influenza in humans. Daneshi *et al*. (2022) hypothesized that the mixing S1b-RBD-expressing mesenchymal stem cell-derived exosomes (47D11 antibody-engineered and previously enriched with remdesivir) be capable of producing effectual delivery to the targeted microenvironment of COVID-19 infection [121]. Additionally, the 47D11 antibody-engineered targeted exosomes can induce immunomodulatory properties, and antiviral actions, abstaining from the entry of severe acute respiratory syndrome-associated coronavirus-2 to ACE (angiotensin-converting enzyme) 2-expressing cells.

### 5.5 Transferosomes

Transferosomes (elastic or deformable liposomes) are elastic vesicular structures containing an edge-activator, and at least one interior aqueous compartment is enclosed by a lipidic bilayer [28]. Its aqueous core is encased by a lipidic bilayer, and produces ultra-deformable vesicular structures, capable of self-optimization and self-regulation [21, 36]. Due to their inherent elasticity, transferosomes can deform and squeeze themselves into skin constrictions or microscopic pores far smaller than the vesicle size without suffering any apparent loss [28]. Edge activators serve as membrane destabilizing agents for the enhancement of the deformability of vesicular membranes [44]. When combined in the ideal ratio with the suitable lipid, transferosomes are made more deformable and ultra-flexible, increasing their permeation capacity [121]. A wide variety of substances can be efficiently transported by transferosomal carriers, regardless of their sizes, structures, molecular weights, polarities, *etc.* Because of these, transferosomes can penetrate the skin pores, much smaller as compared to their diameters and circumvent the main limitation of liposomes. The active chemicals transported *via* transferosomes include proteins, insulin, NSAIDs, interferons, anesthetics, anticancer agents, corticosteroids, and herbal drugs.

Peira *et al*., (2006) reported acyclovir-loaded cationic elastic liposomes for topical delivery to Herpes simplex virus infection [122]. These acyclovir-loaded cationic elastic liposomes were prepared using l-alanine benzyl ester or stearylamine and sucrose monopalmitate (as a deforming agent), which exhibited high acyclovir entrapment efficiency. When applied non-conclusively on the skin of pig ear, skin permeation flux of acyclovir from these acyclovir-loaded cationic elastic liposomes was found comparable to that of the acyclovir-loaded non-elastic cationic liposomes. However, it was found higher as compared to that of acyclovir-loaded non-elastic anionic liposomes or acyclovir suspension (as control). The skin deposition of acyclovir from acyclovir-loaded elastic cationic liposomes was found elevated as compared to that from acyclovir-loaded non-elastic cationic or anionic liposomes and then that from acyclovir-loaded elastic anionic liposomes. Jain *et al*., (2008) developed acyclovir sodium-loaded elastic liposomes for transdermal delivery, which were formulated *via* the conventional rotary evaporation technique [123]. Using confocal laser scanning microscopy, the skin permeation of acyclovir was evaluated and the skin permeation result demonstrated an improved skin permeation of acyclovir from the developed elastic liposomes to the deeper skin layers with a better flux and reduced lag time. In *in vivo* evaluation, the developed elastic liposomes exhibited 105 ± 9.40 ng/ml acyclovir sodium in plasma after 24 h of transdermal application, which was estimated about 4.20 fold as compared to that of the conventional liposomes. Hussain *et al*. (2023) formulated acyclovir-loaded elastic liposomes to control cutaneous infection of herpes and herpes keratitis [114]. They investigated the acyclovir permeation using cultured human EpiDerm, and rat skin. The vesicle size of reformulated acyclovir-loaded was 217 nm. However, the acyclovir permeation from formulated acyclovir-loaded elastic liposomes was not better than acyclovir-loaded ethosomes.

## CONCLUSION AND FUTURE PROSPECTS

Since the nanomedicine technique has been introduced into therapeutic approaches, the possibility of eliminating viral illnesses is increasing. Nanomedicine has produced several novel solutions that have increased treatment efficiency and reasonable success rates. Nanomedicine significantly impacts antiviral therapy because it increases bioavailability, enables targeted drug administration, reduces unfavourable side-effects, lowers the cost of treatment, and generally promotes antiviral therapy. Compared to the conventional treatment procedures, which need higher doses of antiviral medication, and rely on the patients adhering to the antiviral therapy routine, these features are beneficial for the effectiveness of viral illness therapy. Nanomedicine also improves patient compliance because it reduces the length of the treatment regimen and the frequency of doses, making it a more economical strategy. Moreover, early virus diagnosis, preventative therapy, and personalized therapy are all done using nanomedicine.

The utilization of nanovesicles in the delivery of systemic and topical medications has enormous potential for the delivery of antiviral medicines. One benefit of nanovesicles is their painless and non-invasive delivery method. However, nanovesicles as antiviral drug delivery systems still need more study before they can be developed and made commercially available. One challenge is nanotoxicology, which requires a study of nanovesicles' toxicity and bio-elimination properties to ensure safety.

The upcoming developments in nanovesicles for antiviral drug delivery should involve virologists. Their participation is essential for evaluating the risk-benefit ratio for the newly developed antiviral drug delivery formulations. Also, virologists would produce new antiviral compounds to be delivered *via* the nanovesicular carriers. In addition, virologists and nanoscientists would collaborate to maximize their attempts to design and develop more biodegradable, and non-toxic nanovesicular carrier-systems for the effective delivery of antiviral molecules. As a result, future approaches should comprise a comprehensive plan for the commercial production of different nanovesicular carrier-systems using appropriate preparation methods. Last but not least, newly created antiviral nanovesicles should be secure, of superior quality, and effective in treating disease, and they should be made accessible to developing nations. This could only be accomplished by tackling the scientific as well as ethical dilemmas confronting antiviral nanomedicine research.

## Figures and Tables

**Fig. (1):**
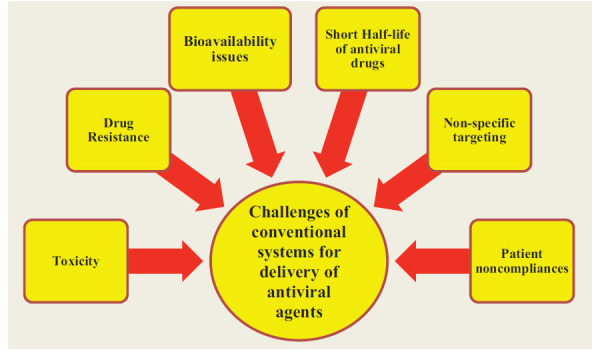
Different challenges of conventional delivery of antiviral drugs.

**Fig. (2):**
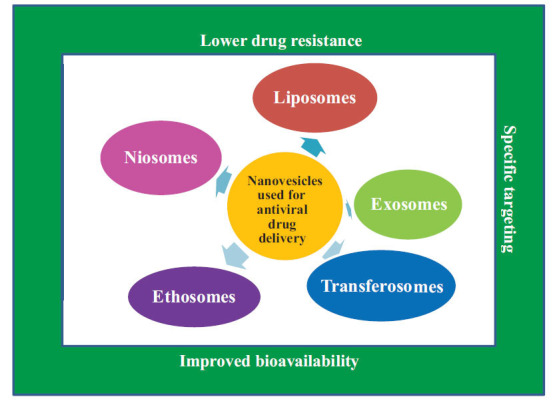
Different nanovesicles for antiviral drug delivery and their potential advantages.

**Table 1:** Different liposomal formulations for antiviral drug delivery.

**Liposomal Formulations**	**Antiviral Drug Loaded**	**Specific Viruses the Loaded Drugs Can Treat, if Mentioned**	**References**
Chitosan/hyaluronic acid film stabilized nanoliposomes to treat coronavirus infection	Remdesivir	Coronavirus	Milkova *et al*., (2023) [72]
Liposomes for improved tissue targeting	Lamivudine	HIV	Godbole *et al*., (2020) [73]
Spray dried liposomes	Lopinavir	HIV	Maniyar *et al*., (2020) [74]
Glycyrrhetinic acid-modified cationic liposomes for targeted HBV infection therapy	Tenofovir	HBV	Yao *et al*., (2020) [75]
Gelatin liposomes to treat HIV infection	Stavudine	HIV	Nayak *et al*., (2017) [76]
Dual drug-loaded liposomes for HIV infection therapy	Enfuvirtide-Protoporphyrin IX	HIV	Figueira *et al*., (2020) [77]
Liposomal drug delivery with glutathione	Efavirenz	HIV	Kenchappa *et al*., (2022) [78]
Liposomal hydrogels for topical vaginal delivery for HIV infection prevention	Emtricitabine and tenofovir disoproxil fumarate	HIV	Faria *et al*., (2019) [79]
Proliposomes for oral delivery	Lopinavir	-	Patel *et al*., (2017) [80]
Functionalized proliposomes for lymphatic targeting	Ritonavir	-	Ahammed *et al*., (2017) [81]
Proliposomal powder for oral delivery	Darunavir	-	Bhusari *et al*., (2020) [82]
Liposomal formulations to treat HIV and AIDS infections	Tenofovir	HIV	Spinks *et al*., (2017) [83]
Aerosolized nanoliposomal carrier for treatment of COVID-19	Remdesivir	Coronavirus	Vartak *et al*., (2021) [84]
Liposomal inhalation for targeted delivery to lungs	Remdesivir	Coronavirus	Li *et al*., (2021) [85]
Liposomes for anti-HIV therapy	Efavirenz	HIV	Kheoane *et al*., (2023) [86]
Liposomes for intranasal systemic delivery	Acyclovir	-	Alsarra *et al*., (2008) [87]

**Table 2:** Different niosomal formulations for antiviral drug delivery.

**Niosomal Formulations**	**Antiviral Drug Loaded**	**Specific Viruses the Loaded Drugs Can Treat, if Mentioned**	**References**
Menthol-containing niosomes	Lopinavir	-	Fayed *et al*., (2022) [97]
Niosomes for treatment of HSV type-1 infection	Acyclovir	HSV	Monavari *et al*., (2014) [98]
Mucoadhesive niosomal gels for AIDS treatment	Tenofovir	HIV	Zidan and Habib (2014) [99]
Niosomes for oral delivery	Tenofovir disoproxil fumarate	Retrovirus	Kamboj *et al*., (2014) [100]
Niosomal dispersion for oral delivery	Ganciclovir	-	Akhter *et al*., (2012) [101]
Nano-gold-loaded mannosylated niosomes containing thermogel system	Efaverinz	HIV-1	Malik *et al*., (2018) [102]
Niosomes for HIV/AIDS treatment	Nevirapine	HIV	Witika and Walker (2019, 2021) [103, 104]

**Table 3:** Different ethosomal formulations for antiviral drug delivery.

**Ethosomal Formulations**	**Antiviral Drug Loaded**	**Specific Viruses the Loaded Drugs Can Treat, if Mentioned**	**References**
Topical ethosomal gel for HSV-1 Infection treatment	Dimethylfumarate	HSV-1	Sicurella *et al*., (2023) [110]
Ethosomes-loaded transdermal patch	Stavudine and lamivudine	-	Gupta *et al*., (2019) [111]
Ethosomes for treatment of HSV-1 infection	Acyclovir	HSV-1	Cortesi *et al*., (2010) [112]
Ethosomes for transdermal delivery	Lamivudine	-	Sudhakar *et al*., (2021) [113]
Ethosomes	Acyclovir	-	Hussain *et al*., (2023) [114]
Binary ethosomes for transdermal delivery	Acyclovir	-	Alshehri *et al*., (2021) [115]

## LIST OF ABBREVIATIONS

HAART: High Antiretroviral Activity Therapy
HIV: Human Immunodeficiency Virus
HLB: Hydrophilic-Lipophilic Balance
HPV: Human Papilloma Virus
HSV: Herpes Simplex Virus
